# Complete resection of a rectus abdominis muscle invaded by desmoid tumors and subsequent management with an abdominal binder: a case report

**DOI:** 10.1186/s13256-018-1575-5

**Published:** 2018-02-07

**Authors:** Tatsuhiko Ogawa

**Affiliations:** Intensive Care Unit, Kochi Health Sciences Center, Ike 2125-1, Kochi City, Kochi Prefecture Japan

**Keywords:** Desmoid tumor, Surgical reconstruction, Rectus abdominis muscle, Pulmonary function

## Abstract

**Background:**

Desmoid-type fibromatosis is characterized by desmoid tumors, which are benign soft tissue tumors that can be locally aggressive but typically do not metastasize. Desmoid tumors can manifest anywhere in the body, and those in the abdominal cavity account for approximately 30 to 50% of all such tumors. Complete resection with free margins has been the standard treatment, but non-surgical therapies have been implemented recently. However, if tumors are strongly invasive and/or persistently recur, radical surgical resection with free margins remains the primary treatment. Unfortunately, radical resection may cause large abdominal defects and hinder reconstruction. Several reports and recommendations have addressed this issue; however, to the best of our knowledge, few reports have described complete resection and the subsequent reconstruction of the rectus abdominis muscle.

**Case presentation:**

A 35-year-old Asian woman presented at our hospital with a chief complaint of abdominal pain. She had abdominal desmoid tumors that required complete resection of her rectus abdominis muscle. Due to necrosis in her own reconstructed tissue, we failed to cover her anterior abdominal wall; thus, we used an abdominal binder as a substitute material to avoid exacerbating the incisional hernia and help her generate intra-abdominal pressure.

**Conclusions:**

This case report may be informative and helpful for the treatment of patients with desmoid tumors, as managing desmoid-type fibromatosis is difficult.

## Background

Desmoid-type fibromatosis is a condition characterized by benign, soft tissue tumors and is regarded as a form of familial adenomatous polyposis (FAP) caused by a germline mutation in the adenomatous polyposis coli (*APC*) gene [1]. These tumors present with local invasion and frequent recurrence even after complete resection; therefore, continuous follow-up and proper intervention are required. Many therapeutic strategies have been used depending on the specific characteristics of the disease [2]. Radical tumor resection with free margins has typically been recommended as the first-line therapy [3]; however, an increasing number of surgeons are using “wait and see” strategies [4–6] because an optimal management strategy has not been determined. There are several case reports [7–9] describing surgical approaches for resecting desmoid tumors when present in the abdominal wall, but the best surgical strategy remains controversial and is dependent on the patient’s background and the surgeon’s preference. Here we present the first reported case of recurrent desmoid tumors that required complete resection of the rectus abdominis muscle and the difficulties experienced during reconstruction, which led to a permanent incisional hernia controlled by an abdominal binder. This case required extensive consideration and a tailor-made treatment. We believe that this case report will be helpful for all medical professionals responsible for treating these difficult problems and can lead to better patient outcomes.

## Case presentation

**Fig. 1:**
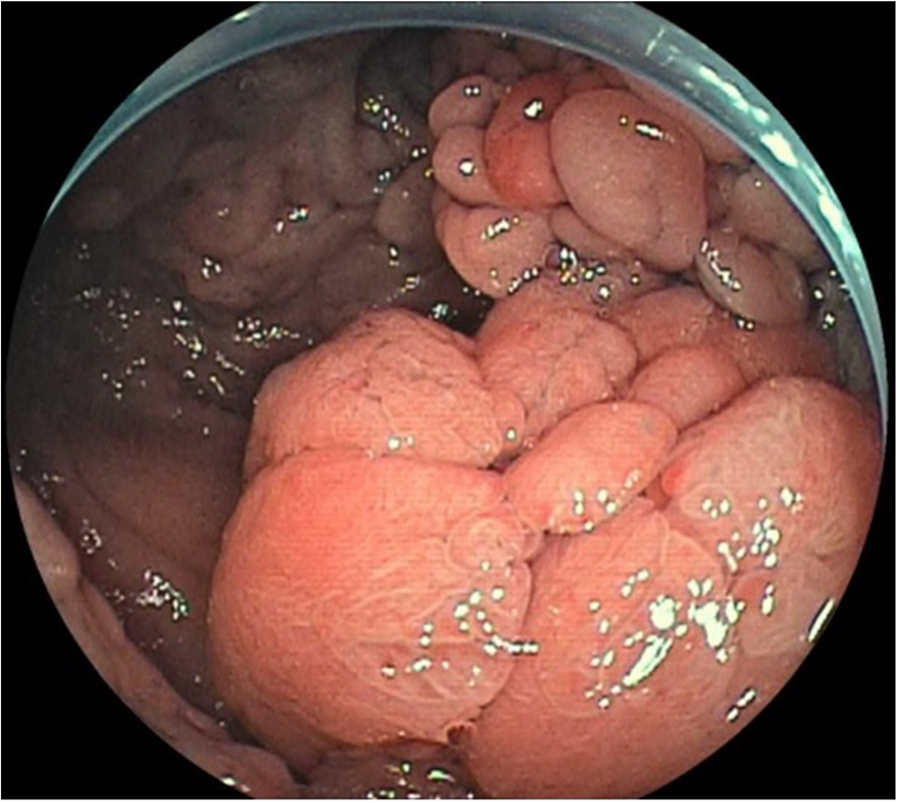
Polyposis observed during the patient’s colonoscopy

A 35-year-old Asian woman presented at our hospital with a chief complaint of abdominal pain. She was diagnosed as having FAP via genetic testing 10 years prior and had undergone multiple endoscopic polypectomies (Fig. 1). She had previously undergone four operations, including multiple resections of desmoid tumors from her abdominal cavity and a total hysterectomy. She had taken tamoxifen as a hormone therapy to inhibit tumor progression [4] and was an obese patient (body mass index, 32 kg/m^2^) who did not smoke tobacco, drink alcohol, or use any illegal substances. She was a homemaker and lived with her husband and her child in an environment with no specific risk factors. However, she had been diagnosed as having a dissociative disorder, for which she had been taking tranquilizers. She delivered her child without any complications. Her mother and one sister also suffered from FAP, and the mother had died from colon cancer caused by FAP.

**Fig. 2:**
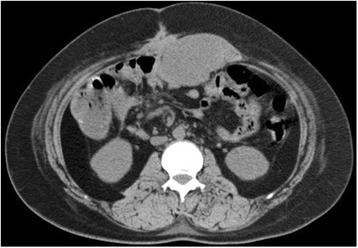
Computed tomography image of the abdominal wall mass

On admission, a solid mass was confirmed in her abdomen by physical examination; however, a neurological examination did not show any abnormalities, and her vital signs were within normal limits as follows: temperature, 36.3 °C; heart rate, 93 beats/minute; blood pressure, 118/80 mmHg; oxygen saturation, 100%; and respiratory rate, 20 breaths/minute. Her initial blood tests did not reveal abnormal results other than slight anemia (hemoglobin, 10.7 g/dL; hematocrit, 35.4%). Preoperative computed tomography (CT) revealed one large mass (approximately 9.3 × 4.3 × 15.0 cm) in her abdominal wall (Fig. 2) and multiple masses in her abdominal cavity. We suspected that these tumors were likely to be recurrent desmoid tumors rather than other soft tissue tumors based on her genetic diagnosis and medical history. The tumors had increased in size and number during the year after her previous surgery, and she was experiencing worsening abdominal pain. Based on the preoperative CT findings, the tumors appeared to be widespread in her rectus abdominis muscle and parts of her colon. Complete surgical resection of the tumors and affected sections of her colon was thought to be the best treatment at this stage to relieve her symptoms and to suppress disease progression. To prepare her for surgery, we administered both epidural and general anesthesia. After the tissue surface was incised, macroscopic observation of the intra-abdominal condition revealed that the tumors were extensively integrated throughout the muscle of her abdominal wall. Almost her entire rectus abdominis muscle as well as some areas of her transverse abdominal muscle were invaded by tumors. We resected the detectable tumors to the greatest possible extent, which resulted in a long wound from her xiphoid process to the suprapubic point; the resection also resulted in the complete removal of her rectus abdominis muscle, including the anterior and posterior sheaths and parts of her transverse abdominal muscle. In addition, her transverse colon strongly adhered to some tumors; thus, we performed a partial colectomy. The typical appearance of spindle cell bundles in the stroma [1] was confirmed by postoperative pathological examination of the resected specimen. Immunohistochemistry revealed that the nuclei of the tumor cells were positive for β-catenin, which is also characteristic of this tumor [5]. Unfortunately, microscopic, positive surgical margins were found in the resected specimen. However, following the European Consensus [5], we considered that reassessing overall disease management and preserving all remaining bodily functions were of utmost importance.

**Fig. 3:**
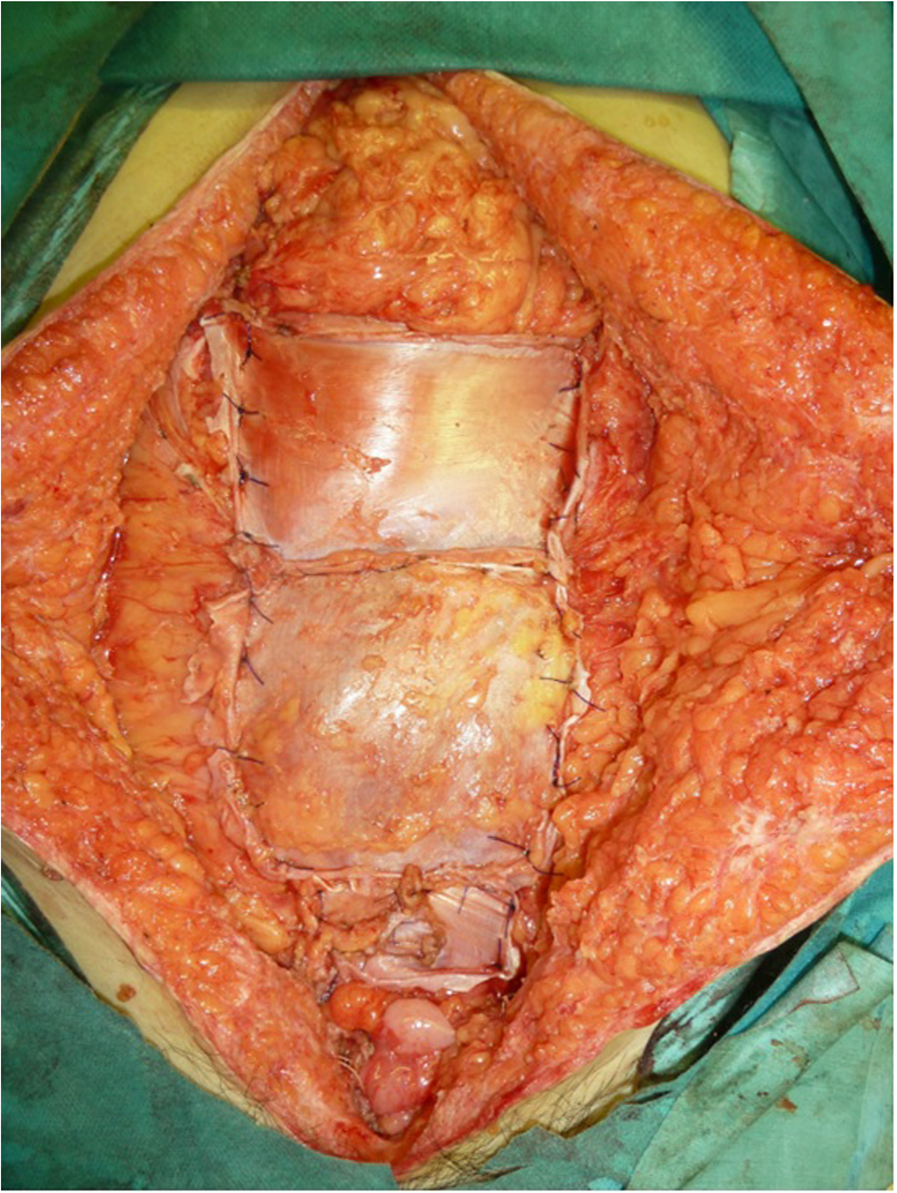
Abdominal defect and the transplanted fascia lata

Complete resection of her rectus abdominis muscle resulted in a large defect in her anterior abdominal surface, and reconstruction was required to cover it. Desmoid tumors frequently recur following trauma, previous operations, or tissue stimulation [1, 3]. Based on her medical history, these tumors had persistently recurred and showed strong invasive tendencies. In addition, unavoidable colectomy procedures could introduce intestinal microorganism contamination. Therefore, we wanted to avoid stimulating her abdominal tissues and organs. To reduce the risk of recurrence, we did not use prosthetic materials, including biological mesh. We also avoided performing intraoperative abdominal wall maneuvers and used her own tissues to reconstruct her abdominal wall to reduce the risk of infection [10]. To cover the broad defect, autograft tissue was peeled from both sides of her fascia lata following our plastic surgeons’ usual technique and other recommendations [11]. The large autograft was transplanted into her abdominal defect (Fig. 3). The operation was successful, and after the surgery, her abdominal wall consisted of only skin, subcutaneous tissue, and the autograft. In addition, we used an abdominal binder to cover the abdominal surface, minimize unwanted pressure on her abdomen and avoid the protrusion of abdominal organs through the autograft, as well as to help her maintain a proper neutral posture, which was made difficult owing to the loss of muscle. We were unable to evaluate the exact influence of the abdominal surgery on her pulmonary function. Muscle loss and the abdominal binder prevented full expiration and inspiration. Furthermore, the wound from her xiphoid process to the suprapubic point could have caused severe pain and prevented her from breathing fully after the operation despite the application of epidural anesthesia.

Because of these circumstances, we did not extubate her in the operating room and decided to continue mechanical ventilation until the inflammatory reactions were sufficiently reduced and the autograft could stabilize in her body. Several days after the operation, the ratio of the partial pressure of oxygen in arterial blood to the inspired oxygen fraction (PaO_2_/FiO_2_) was less than 200, and we concluded that she could not breathe effectively on her own without mechanical support; therefore, we continued mechanical ventilation. Three days after the operation, she developed a high fever, and blood tests showed a strong inflammatory response. Moreover, non-malodorous liquid was oozing from the operative wound. Therefore, we drained the surgical site of several hundred milliliters of exudate. Laboratory testing of liquid samples did not reveal any obvious signs of infection. A subsequent CT examination revealed an accumulation of fluid in her abdominal cavity where the autograft had been placed, with only a small portion remaining. We thus considered that most of the autograft had become necrotic and disintegrated, which had caused the strong inflammatory reactions and the high fever.

After the breakdown of the autograft, our patient developed a large incisional hernia from her xiphoid process to the suprapubic point. There was no muscle in her anterior abdominal wall, and it comprised only dermis and subcutaneous tissue.

We continued mechanical ventilation along with respiratory rehabilitation; however, she was accidentally extubated on postoperative day 6. We considered reintubating her, but she could spontaneously breathe without dyspnea while supported by an oxygen mask.

Blood gas sampling did not reveal any other unfavorable results, such as the progression of acidosis or an increase in lactate; in addition, her PaO_2_/FiO_2_ ratio was within normal limits. Therefore, we decided not to reintubate her, and we continued to provide oxygen support under strict observation.

We were concerned that the abdominal binder might cause extra negative pressure on her abdominal wall during inspiration, but she was able to breathe deeply with sufficient vital capacity (VC). In addition, the abdominal binder helped prevent exacerbation of the incisional hernia through the abdominal defect.

She successfully recovered and did not require any additional surgical interventions. However, owing to muscle loss, she could not tolerate any strain during ordinary daily activities. She could not sit up from a supine position and instead had to rise from a lateral position. In addition, she could not exhale fully or defecate effectively. On the other hand, the abdominal binder was useful for maintaining her posture, protecting her abdominal organs, and preventing any protrusions through the abdominal defect despite the permanent presence of an incisional hernia. She did not feel any discomfort from wearing the abdominal binder for most of the day. Her spirometry results showed the following changes between her preoperative and postoperative conditions with the abdominal binder: VC decreased from 1.95 L to 1.75 L, forced VC (FVC) decreased from 1.95 L to 1.75 L, forced expiratory volume in 1 second (FEV1) decreased from 1.66 L to 1.54 L, the FEV1/FVC ratio increased from 0.85 to 0.88, and peak expiratory flow (PEF) decreased from 4.51 L/second (78.0%) to 4.34 L/second (75.3%). Thus, she exhibited only a mildly restrictive ventilation disorder and a decreased ability to exhale. Overall, she adjusted well to everyday life without her rectus abdominis muscle because of the abdominal binder.

After undergoing surgery, her remaining desmoid tumors gradually increased in size and recurred in another area of the abdominal soft tissue, which caused worsening abdominal pain. To prevent the tumors from invading the visceral organs and avoid exacerbating the difficulties of repeated surgery, we performed simple tumor resections twice during a 1-year follow-up, which resulted in the removal of only a portion of her soft tissue. However, we successfully avoided colectomy and injury to other organs, and there was no further deterioration of pulmonary function or exacerbation of the incisional hernia owing to the binder. Positive margins were confirmed in the resected specimens; however, no practical harmful influences on her body have been confirmed after a postoperative period of 6 months. We will continue to meticulously follow this patient and discuss surgical indications, including potential early interventions and predicted worsening of her bodily functions, during each visit.

## Discussion and conclusions

There have been many reports about the reconstruction of abdominal wall defects [7–12]; however, this is the first case report of abdominal desmoid tumors that required complete resection of the rectus abdominis muscle and led to a subsequent permanent incisional hernia controlled by an abdominal binder. We present this case report for guidance in the management of this complicated condition.

Desmoid tumors are benign tumors, but their local invasive nature and high rates of recurrence present surgeons with difficult choices and require complicated treatments [2–5], including surgical tumor resection, radiation therapy, hormonal therapy, and administration of non-steroidal anti-inflammatory drugs (NSAIDs). Chemotherapy is usually reserved for patients with significant symptoms who fail to respond to more benign interventions such as NSAIDs and tamoxifen [4].

Desmoid tumors may occur at sites of previous trauma [1, 3] and are suggested to be related to hormonal changes in females [1]. Early surgical intervention, including radical resection of tumors with clear margins, has been the standard surgical treatment [2]. However, other surgeons have recently suggested that conservative therapy with continuous follow-up is safer than a radical surgical approach [4–6]. The authors of the UK guidelines [4] referred to the recent European Consensus [5] and stated that watchful waiting should be the standard first-line option.

The tumors in our patient had progressively developed in size and number, and she began experiencing worsening pain within 1 year after the previous tumor resection. Therefore, we concluded that surgical intervention was inevitable and that a conservative approach would not be applicable in this situation.

Unfortunately, positive margins around the tumors were confirmed postoperatively. The European Consensus statement [5] recommends the “wait and see” approach based on previous reports [13–16] that have described recurrence despite negative margins and no recurrence despite positive margins; therefore, we prioritized our patient’s quality of life.

Several reports [7–9] have described cases of abdominal wall reconstruction after wide resection; however, there are currently no definitive guidelines for this type of reconstruction. Surgeons have used prosthetic meshes or autografts such as those from the fascia lata or the latissimus dorsi muscle [7–9, 12].

Based on our patient’s history, we did not want to stimulate her immune response with any prosthetic materials, including a biological mesh, to deter recurrence [1, 3]. In addition, judging from the intraoperative condition of her abdominal cavity, the tumors were thought to strongly adhere to her colon and a colectomy to resect these tumors was inevitable. This approach is associated with a risk of infection and based on a previous report [10] we concluded that this risk contraindicated the use of synthetic mesh. For this reason, we used her own tissues to reconstruct her abdominal wall. This approach has been recommended by another author because autografts are associated with fewer complications, including graft infection, graft degradation, and possible local or systemic rejection [11].

Unfortunately, the autograft became necrotic in this patient, and the transplantation failed. We could not definitively ascertain the cause of tissue necrosis; however, during the procedure, we had to radically resect the abdominal wall muscles with broad free margins (approximately several centimeters), and this might have seriously damaged her tissues and led to the production of excessive exudate. In addition, we had to administer fluids to maintain cardiovascular circulation during surgery and this might have caused a volume overload. As a result, it is possible that ascites and exudate had accumulated in her abdominal cavity and subsequently prevented the lining of the fascia lata from adhering to the peripheral abdominal wall and organs. This condition might have impaired microvascular circulation in the peripheral tissues and caused necrosis of the autograft. Ultimately, her abdomen was covered only by the dermis and subcutaneous tissue, and we discussed the necessity of repair with our patient. Previous reports [1, 3] have indicated that a repeat operation on our patient’s abdomen and the insertion of artificial materials into her abdominal cavity could increase the possibility of recurrence and formation of a new malignant tumor. On the other hand, without her abdominal wall, she would have to spend the rest of her life with a permanent incisional hernia. To avoid exacerbating this condition, she would have to constantly wear an abdominal binder, which could impair pulmonary function. However, considering her background, we thought that the risk of recurrence and novel cancer development induced by the artificial material was much greater than the risk of other complications. Furthermore, our patient did not express any dyspnea or discomfort with the abdominal binder. Therefore, we decided to leave the abdominal condition as it was after the failed transplantation and continued to use the external abdominal binder with meticulous follow-up. We were concerned about the influence of the binder on her pulmonary function, but she did not exhibit any serious symptoms with this extra pressure on her abdomen. Several other reports have described the influence of abdominal binders on pulmonary function. For example, Arici *et al*. [17] stated that there were no significant differences in the mean FEV1, FVC, and FEV1/FVC values (*p* > 0.05) between patients who underwent major abdominal surgery with and without abdominal binders, and this research group hypothesized that the impact of using an abdominal binder for pain control positively affected the efficacy of regular pulmonary exercises. They also concluded that the use of an abdominal binder does not have a clinically meaningful negative impact on pulmonary function. Rothman *et al*. performed a systematic review [18] and found no evidence that abdominal binders reduced or improved postoperative pulmonary function and that these binders could be used without compromising pulmonary function. However, this conclusion did not apply to our patient. Without her rectus abdominis muscle, she could not strain herself and exhibited related symptoms. Her spirometry results showed worsened expiratory capacity as evidenced by her FEV1, FVC, and PEF data. In addition, a slightly worsened VC was confirmed. Being a non-tobacco smoker and a relatively healthy young patient despite recurrent surgeries might have contributed to her relatively unimpaired pulmonary function. The slightly worsened expiratory results might have been due to the lack of her rectus abdominis muscle, which is used during expiration. The binder might have helped her exhale by providing intra-abdominal pressure, but it also might have inhibited full inspiration, as indicated by the worsened VC.

Unfortunately, positive margins of the remaining desmoid tumors gradually increased in size, and recurrence occurred repeatedly in another area of her abdominal soft tissue. Meticulous follow-up and discussion of optimal therapies at each visit, including surgical intervention and the appropriate use of an abdominal binder, may help maintain the stability of our patient’s condition and avoid further deterioration of all other remaining bodily functions and exacerbation of the incisional hernia.

In conclusion, desmoid tumors cause many difficult clinical problems that require multidisciplinary approaches. The best management of desmoid tumors must be determined based on the patients’ background and condition. Wide resection of the abdominal wall (including complete resection of the rectus abdominis muscle) and covering the defect using only the dermis and subcutaneous tissue could subsequently cause an incisional hernia. However, in such complicated cases, the use of abdominal binders could lessen the exacerbation of the incisional hernia, protect abdominal organs, and maintain proper posture, resulting in a relatively high quality of life.

## Abbreviations

CT: Computed tomography
FAP: Familial adenomatous polyposis
FEV1: Forced expiratory volume in 1 second
FVC: Forced vital capacity
NSAIDs: Non-steroidal anti-inflammatory drugs
PaO_2_/FiO_2_ ratio: Ratio of the partial pressure of oxygen in arterial blood (PaO_2_) to the inspired oxygen fraction (FiO_2_)
PEF: Peak expiratory flow
VC: Vital capacity
